# Cigarette smoking and personality: interrogating causality using Mendelian randomisation

**DOI:** 10.1017/S0033291718003069

**Published:** 2018-10-25

**Authors:** Hannah M. Sallis, George Davey Smith, Marcus R. Munafò

**Affiliations:** 1 MRC Integrative Epidemiology Unit at the University of Bristol, Bristol, UK; 2 UK Centre for Tobacco and Alcohol Studies, School of Psychological Science, University of Bristol, Bristol, UK; 3 Population Health Sciences, Bristol Medical School, University of Bristol, Bristol, UK; 4 Centre for Academic Mental Health, Population Health Sciences, Bristol Medical School, University of Bristol, Bristol, UK

**Keywords:** Extraversion, Mendelian randomisation, neuroticism, personality traits, smoking behaviours

## Abstract

**Background:**

Despite the well-documented association between smoking and personality traits such as neuroticism and extraversion, little is known about the potential causal nature of these findings. If it were possible to unpick the association between personality and smoking, it may be possible to develop tailored smoking interventions that could lead to both improved uptake and efficacy.

**Methods:**

Recent genome-wide association studies (GWAS) have identified variants robustly associated with both smoking phenotypes and personality traits. Here we use publicly available GWAS summary statistics in addition to individual-level data from UK Biobank to investigate the link between smoking and personality. We first estimate genetic overlap between traits using LD score regression and then use bidirectional Mendelian randomisation methods to unpick the nature of this relationship.

**Results:**

We found clear evidence of a modest genetic correlation between smoking behaviours and both neuroticism and extraversion. We found some evidence that personality traits are causally linked to certain smoking phenotypes: among current smokers each additional neuroticism risk allele was associated with smoking an additional 0.07 cigarettes per day (95% CI 0.02–0.12, *p* = 0.009), and each additional extraversion effect allele was associated with an elevated odds of smoking initiation (OR 1.015, 95% CI 1.01–1.02, *p* = 9.6 × 10^−7^).

**Conclusion:**

We found some evidence for specific causal pathways from personality to smoking phenotypes, and weaker evidence of an association from smoking initiation to personality. These findings could be used to inform future smoking interventions or to tailor existing schemes.

## Introduction

There is a well-documented association between smoking behaviours and personality traits such as neuroticism and extraversion (Terracciano and Costa, 2004; Malouff *et al*., 2006; Munafò *et al*., 2007; Hakulinen *et al*., 2015), and with associated mental health outcomes such as major depressive disorder (MDD) (Munafò and Araya, 2010; Fluharty *et al*., 2017). However, given that much of these data come from observational studies, it is difficult to establish whether these relationships are causal. It is possible that the observed associations could be due to confounding, and if a true causal relationship does exist, the direction of effect is unknown.

Understanding these relationships is important for public health and policy. The World Health Organisation (WHO) now recognises smoking as one of the leading modifiable risk factors for disability, disease and death (World Health Organisation, 2002). As a result, if it were possible to unpick the association between personality and smoking, it may be possible to develop more tailored smoking interventions, which could lead to both improved uptake and efficacy. For example, existing smoking cessation schemes could be tailored according to personality traits (Cherry and Kiernan, 1976), with individuals allocated a different number of sessions or intensity of intervention according to their level of neuroticism.

Neuroticism and extraversion are two of the main components of personality. The former reflects emotional instability, stress vulnerability and proneness to anxiety (Kendler *et al*., 1993). Higher neuroticism has been linked to anxiety and MDD, with some evidence of shared genetics and a causal link between neuroticism and MDD onset (Neale *et al*., 2005; Gale *et al*., 2016). Although levels of neuroticism are higher among smokers, the evidence that neuroticism is linked with smoking initiation is inconsistent, with one meta-analysis suggesting that neuroticism is linked with relapse to smoking among former smokers (Hakulinen *et al*., 2015) rather than smoking initiation. In contrast, extraversion is characterised by tendencies such as liveliness and assertiveness of an individual and the level of ease and enjoyment of social interactions (Kendler *et al*., 1993; van den Berg *et al*., 2016). There is some suggestion in the literature that high levels of extraversion are associated with greater rates of smoking initiation, and lower rates of smoking cessation (Hakulinen *et al*., 2015).

**Fig. 1.**
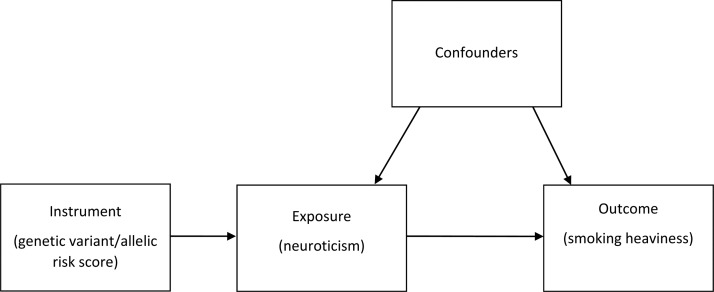
Directed acyclic graph illustrating Mendelian randomisation. In this model, allelic risk scores associated with neuroticism are calculated and used to assess the association of neuroticism with smoking heaviness.

With traditional epidemiological methods, it is difficult to establish causality using observational data due to issues such as reverse causation and confounding. Mendelian randomisation (MR) is a technique that enables us to assess causal effects in observational datasets using genetic instrumental variables (Sallis *et al*., 2014). MR can be thought of as analogous to a randomised controlled trial, with the genetic variants used as proxies or markers of randomisation into a group that later goes on to encounter more of a risk factor, such as smoking (Fig. 1). The MR principle relies on approximations of Mendel's first and second laws of segregation and independent assortment (Davey Smith, 2011). Assuming the genetic variants are not associated with the outcome other than through the risk factor of interest, we can make inferences about the causal direction of any association between the risk factor and the outcome (Davey Smith and Ebrahim, 2003). If the underlying assumptions of MR are satisfied, the resulting effect estimates should be free from the problems of confounding and reverse causality to which observational epidemiology is prone (Sallis *et al*., 2014), although interpretation of such estimates requires careful consideration (Holmes *et al*., 2017).

Previous work using MR have yielded no strong evidence of a causal relationship in the direction of smoking to depression (Taylor *et al*., 2014). We therefore hypothesised that if a causal association with neuroticism exists, it is more likely to act from neuroticism to smoking. Recent genome-wide association studies (GWAS) have identified variants robustly associated with a number of smoking phenotypes (Tobacco and Genetics Consortium, 2010) and with personality traits (Okbay *et al*., 2016; Lo *et al*., 2017). Here we use these publicly available GWAS summary statistics in addition to individual-level data from UK Biobank (UKB) (Sudlow *et al*., 2015) in a bidirectional analysis to investigate whether there appears to be a causal link between smoking and personality.

## Methods and measures

### Genetic instruments

#### Smoking behaviour

Three smoking behaviours were investigated: initiation, heaviness and cessation. For each of the smoking phenotypes, a single variant identified by the Tobacco and Genetics (TAG) consortium (2010) was used as a genetic instrument for this behaviour. These were as follows: the rs6265 variant in the *BDNF* gene identified for smoking initiation, the rs16969968 variant in the *CHRNA5* gene for smoking heaviness among past and current smokers and the rs3025343 variant in the *DBH* gene for smoking cessation. For each phenotype, the effect allele was that which corresponded to an increase in the relevant smoking behaviour.

#### Personality

Eleven independent variants associated with neuroticism were reported by Okbay *et al*. (2016). Of the original variants, five of these were unavailable in the TAG smoking data. Proxies were identified for four of these single-nucleotide polymorphisms (SNPs) using SNIPA (Arnold *et al*., 2015) (*r*^2^ > 0.85). A complete list of variants included in the neuroticism instrument can be found in online Supplementary Table S1. Five independent variants associated with extraversion were identified using data from the Genetics of Personality Consortium (GPC) and 23andMe (Lo *et al*., 2017). Of these variants, three variants were unavailable in the TAG summary statistics. Using SNIPA, we identified a proxy for one of these variants (online Supplementary Table S1). There was no overlap between the variants included in any of the smoking or personality instruments.

The amount of variance explained by each of these instruments in UKB is reported in online Supplementary Table S2.

### Data sources

Summary statistics from several GWASs were used for two-sample MR analyses, while individual-level data from UKB were used for one-sample analyses.

#### GWAS summary statistics

Publicly available summary statistics are available for recent genome-wide analyses of both smoking phenotypes and personality traits. GWAS summary statistics for smoking initiation, cessation and heaviness were published by the TAG consortium (2010). Summary statistics for personality traits were taken from two recent GWASs of neuroticism and extraversion (Okbay *et al*., 2016; Lo *et al*., 2017).

#### UK Biobank

UKB has collected phenotypic information on around 500 000 participants, with genotyping available on approximately 337 106 unrelated Europeans, exclusion criteria and quality control measures are described in detail elsewhere (Bycroft *et al*., 2017; Mitchell *et al*., 2017). An interim release of genetic data was made available in 2015 for a subset of the cohort. This subset was included in the neuroticism GWAS and contained approximately 114 780 European individuals (Sudlow *et al*., 2015).

Smoking status was defined as ever (consisting of current and former smokers) or never smoker according to responses given at the initial assessment visit in UKB. Smoking heaviness was derived for former and current smokers based on responses to ‘number of cigarettes currently smoked daily’ at the initial assessment. For former smokers, this question related to number of cigarettes previously smoked on a daily basis.

Neuroticism scores were derived from a number of neurotic behaviour domains measured at the initial assessment visit. These scores were externally derived by Smith *et al*. (2013) and are available for use by researchers accessing the UKB resource. Scores range from 0 to 12 with a higher score corresponding to a greater number of neurotic behaviours. There was no direct measure of extraversion in UKB, so analyses of this trait were restricted to those using the genetic instrument for extraversion.

Unweighted polygenic risk scores for neuroticism and extraversion were calculated for each individual in UKB based on the number of copies of each risk allele carried by an individual; this ranged from 0 to 2 for each SNP. The neuroticism risk score ranged from 1 to 19 and corresponded to the number of neuroticism increasing alleles per individual. A risk score was calculated similarly for extraversion and ranged from 0 to 6. Although weighted scores can give a more precise effect estimate, the neuroticism GWAS included the interim release of UKB within the discovery sample (Okbay *et al*., 2016; Wray *et al*., 2018). Risk scores should use weightings derived from independent samples to avoid introducing bias into the effect estimates (Hartwig and Davies, 2016).

### Statistical analysis

#### Genetic correlation

In a first step, GWAS summary statistics were used to estimate the genetic correlation of smoking initiation with both neuroticism and extraversion. LD score regression was performed (without constraining the intercept) using the GWAS summary statistics to assess the amount of genetic overlap between the two traits. In order to estimate genetic correlation between personality measures and additional smoking phenotypes of smoking heaviness and cessation, summary statistics for the personality GWAS would need to be stratified by smoking status. Although the original GWAS summary statistics were not available stratified by smoking status, it was possible to estimate these genetic correlations using the individual-level data available in UKB. Genome-wide complex trait analysis (GCTA) software (Yang *et al*., 2011) was used to estimate genetic correlation for each smoking phenotype using data from UKB.

#### Two-sample MR using summary statistics

Bidirectional two-sample MR analyses were performed using the genetic instruments described above. Effect estimates and standard errors (s.e.s) were extracted for each variant from the relevant GWAS results and used to estimate inverse variance weighted (IVW) effect estimates. For the neuroticism instrument which incorporated multiple SNPs, we performed a number of sensitivity analyses. Effect estimates and s.e.s were extracted from the original GWAS results as described above and MR-Egger (Bowden *et al*., 2015) and weighted median regression (Bowden *et al*., 2016) approaches were performed. These are complementary approaches: MR-Egger calculates effect estimates adjusted for horizontal pleiotropy (under the assumption that other conditions of MR-Egger are not violated) (Bowden *et al*., 2015), while weighted median calculates effects which are robust as long as 50% or more of the statistical weight within an analysis comes from valid instruments (Bowden *et al*., 2016). We also calculated Rücker's Q to investigate heterogeneity in the SNP-exposure associations (Bowden *et al*., 2017). Neuroticism and extraversion GWAS results were not stratified by smoking status. As a result, when using summary statistics, analyses in the direction of smoking to personality were restricted to smoking initiation only.

#### One-sample analyses using individual-level data

Further analyses were performed using data from UKB. These analyses estimated the association between the genetic instrument and the outcome as there was no measure of extraversion in the UKB sample, and the neuroticism GWAS included data from UKB which could bias the two-sample IVW estimates. Within UKB, it was possible to stratify participants according to smoking status. Therefore, in addition to smoking initiation, we also investigated the association between both smoking heaviness and cessation with personality. These analyses were adjusted for the top 10 principal components as well as genotype array.

#### Sensitivity analyses

We restricted analyses using the neuroticism risk score to participants who were not included in the interim release of the genetic data, and who were therefore not included in the discovery sample. We also calculated IVW estimates in UKB using the neuroticism instrument for comparison with the effect estimates from the two-sample analysis.

## Results

### Genetic correlation

LD score regression using summary statistics from the TAG consortium GWAS of smoking initiation and the Okbay *et al*. neuroticism GWAS suggested evidence of a modest genetic correlation between the two traits (*r*_G_ = 0.124, s.e. = 0.05, *p* = 0.008). There was also evidence of a larger genetic correlation between extraversion and smoking initiation (*r*_G_ = 0.288, s.e. = 0.01, *p* = 0.001; Table 1). We used GCTA software to calculate genetic correlation using individual-level data from UKB. There was evidence of genetic correlation between neuroticism and smoking heaviness among both current (*r*_G_ = 0.248, s.e. = 0.12, *p* = 0.013) and former smokers (*r*_G_ = 0.220, s.e. = 0.06, *p* = 1.8 × 10^−5^). We found evidence of a negative genetic correlation between smoking cessation and neuroticism (*r*_G_ = −0.314, s.e. = 0.15, *p* = 0.002; Table 1).

**Table 1.** Genetic correlation between smoking phenotypes and personality traits using GWAS summary statistics and individual-level data from UK Biobank

	*r*_G_ (s.e.)	*p* value
Neuroticism and smoking initiation^a^	0.124 (0.05)	0.008
Extraversion and smoking initiation^a^	0.288 (0.01)	0.001
Neuroticism and smoking heaviness (current smokers)^b^	0.248 (0.12)	0.013
Neuroticism and smoking heaviness (former smokers)^b^	0.220 (0.06)	1.8 × 10^−5^
Neuroticism and smoking cessation^b^	−0.314 (0.15)	0.002

a Genetic correlation estimates for smoking initiation were generated using GWAS summary statistics.

b Estimates for smoking heaviness and cessation were generated using individual-level data from UK Biobank.

### Observational association between neuroticism and smoking behaviours

Data on smoking status and neuroticism were available from UKB. A total of 389 770 participants had data available on both smoking and neuroticism, with genotyping available on 273 516 of these after applying QC measures. There was strong evidence of an observational association between neuroticism and smoking status in both the entire UKB sample and when restricting to those with genotyping data. Mean neuroticism scores were higher among former (4.22, s.d. = 3.3) and current smokers (4.66, s.d. = 3.5) than non-smokers (3.89, s.d. = 3.2, *p* < 0.001). We found evidence of an association between neuroticism and cigarettes smoked per day with heavier smokers reporting higher neuroticism scores (*β* = 0.02, 95% CI 0.02–0.03, *p* < 0.001).

### Effects of smoking on personality traits

#### Neuroticism

We first used two-sample MR to investigate the effect of smoking initiation on personality using publicly available GWAS summary statistics. This found no clear evidence of an effect of smoking initiation on neuroticism when using rs6265 as an instrument for smoking initiation (*β* = −0.032, 95% CI −0.16 to 0.09, *p* = 0.617; Table 2). A one-sample approach was also used to investigate the association between each smoking behaviour and neuroticism in UKB. This found weak evidence of an effect, with each copy of the smoking initiation risk allele associated with a lower neuroticism score (*β* = −0.023, 95% CI −0.045 to −0.001, *p* = 0.037; Table 2).

**Table 2.** Effect of smoking on personality traits using one- and two-sample MR

Exposure	Outcome	Sample	Instrument	Smoking status	*β*^a^	95% CI	*p* value	*N*
Smoking initiation	Neuroticism	GWAS summary statistics	rs6265	All	−0.032	−0.16 to 0.09	0.617	170.911
		UK Biobank		All	−0.023	−0.045 to −0.001	0.037	274 230
	Extraversion	GWAS summary statistics	rs6265	All	0.198	−0.03 to 0.42	0.084	63 030
		UK Biobank			–	–	–	–
Smoking heaviness	Neuroticism	UK Biobank		Non-smokers	0.004	−0.02 to 0.03	0.745	149 960
				Former smokers	0.011	−0.02 to 0.04	0.472	96 674
				Current smokers	0.038	−0.02 to 0.10	0.234	26 882
Smoking cessation	Neuroticism	UK Biobank		Non-smokers	0.004	−0.03 to 0.04	0.822	149 960
				Ever smokers	0.029	−0.01 to 0.07	0.161	123 556

a Two-sample results reported using GWAS summary statistics refer to the causal effect of smoking behaviour on a personality trait. UK Biobank results are effect sizes of the genetic variants for smoking behaviours on personality traits.

The genetic variant rs16969968 was used as a proxy for smoking heaviness in UKB. Despite strong evidence of an observational association between smoking heaviness and neuroticism, we found no robust evidence of a causal effect of the rs16969968 variant for smoking heaviness on neuroticism among either former or current smokers (Table 2). Using the rs3025343 variant as a proxy for smoking cessation found no strong evidence of an effect of smoking cessation on neuroticism (*β* = 0.029, 95% CI −0.01 to 0.07, *p* = 0.161; Table 2) in UKB.

#### Extraversion

When using two-sample MR to look at the association between extraversion and smoking, there was some indication that smoking initiation was associated with greater extraversion (*β* = 0.198, 95% CI −0.03 to 0.42, *p* = 0.084; Table 2), although the evidence for this was weak. There was no relevant measure of extraversion in UKB, so we were unable to look at the effect of the genetic variant for smoking initiation on extraversion using individual-level data.

### Effects of personality traits on smoking

#### Smoking initiation

Two-sample MR using summary statistics found no clear evidence for an effect of neuroticism on smoking initiation either when using an IVW approach (OR 1.165, 95% CI 0.71–1.91, *p* = 0.499; Table 3) or when performing sensitivity analyses (online Supplementary Tables S3 and S4). Among the UKB participants, there was no robust evidence for an effect of the neuroticism risk score on smoking initiation (OR 1.000, 95% CI 0.997–1.003, *p* = 0.980; Table 3). When looking at the effect of extraversion on smoking initiation, we found no strong evidence of an effect when using a two-sample approach (OR 1.733, 95% CI 0.37–8.23, *p* = 0.268). However, this may be due to a lack of power. The direction of effect was consistent within the UKB sample, where there was strong evidence of an association. Each additional extraversion allele was associated with 1.5% higher odds of ever smoking (OR 1.015, 95% CI 1.01–1.02, *p* = 9.6 × 10^−7^; Table 3).

**Table 3.** Effect of personality traits on smoking using one- and two-sample MR

Exposure	Outcome	Sample	Instrument	Smoking status	OR^a^	95% CI	*p* value	*N*
Neuroticism	Smoking initiation	GWAS summary statistics	IVW	All	1.165	0.71–1.91	0.499	74 035
		UK Biobank	Unweighted score	All	1.000	0.997–1.003	0.980	318 985
Extraversion	Smoking initiation	GWAS summary statistics	IVW	All	1.733	0.37–8.23	0.268	74 035
		UK Biobank	Unweighted score	All	1.015	1.01–1.02	9.6 × 10^−7^	332 596
					*β*^a^	95% CI	*p* value	*N*
Neuroticism	Smoking heaviness	GWAS summary statistics	IVW	Ever smokers	0.050	−4.11 to 4.21	0.979	38 181
		UK Biobank	Unweighted score	Former smokers	−0.018	−0.05 to 0.02	0.315	70 909
				Current smokers	0.068	0.02–0.12	0.009	21 449
Extraversion	Smoking heaviness	GWAS summary statistics	IVW	Ever smokers	0.017	−16.33 to 16.37	0.997	38 181
		UK Biobank	Unweighted score	Former smokers	−0.021	−0.08 to 0.04	0.519	73 967
				Current smokers	−0.038	−0.13 to 0.05	0.419	22 340
					OR^a^	95% CI	*p* value	*N*
Neuroticism	Smoking cessation	GWAS summary statistics	IVW	Ever smokers	0.797	0.43–1.47	0.423	41 278
		UK Biobank	Unweighted score	Ever smokers	0.997	0.99–1.00	0.272	144 119
Extraversion	Smoking cessation	GWAS summary statistics	IVW	Ever smokers	0.586	0.07–4.76	0.387	41 278
		UK Biobank	Unweighted score	Ever smokers	0.989	0.98–1.00	0.057	150 294

a Two-sample results reported using GWAS summary statistics refer to the causal effect of personality traits on smoking behaviours. UK Biobank results are effect sizes of the genetic instruments for personality traits on smoking behaviours.

#### Smoking heaviness

Using an IVW approach, we found no clear evidence for an effect of neuroticism on smoking heaviness (*β* = 0.050, 95% CI −4.11 to 4.21, *p* = 0.979; Table 3). However, MR-Egger suggested some evidence of horizontal pleiotropy (*β* = −0.500, 95% CI −0.93 to −0.07, *p* = 0.026; online Supplementary Tables S3 and S4) and the bias-adjusted estimate suggested some evidence of an association between neuroticism and greater smoking heaviness (*β* = 22.55, 95% CI 2.82–42.27, *p* = 0.027). We also looked at this association in UKB when stratifying according to smoking status and found some evidence of an association. Among current smokers, the neuroticism risk score was associated with greater smoking heaviness (*β* = 0.068, 95% CI 0.02–0.12, *p* = 0.009; Table 3). In this analysis, each additional neuroticism risk allele was associated with smoking an extra 0.07 cigarettes per day. We found no robust evidence for an effect of extraversion on smoking heaviness either when using a two-sample MR approach (*β* = 0.017, 95% CI −16.33 to 16.37, *p* = 0.997; Table 3) or when stratifying on smoking status and investigating this association in UKB. This remained the case among both former (*β* = −0.021, 95% CI −0.08 to 0.04, *p* = 0.519) and current smokers (*β* = −0.038, 95% CI −0.13 to 0.05, *p* = 0.419; Table 3).

#### Smoking cessation

When using two-sample MR with summary statistics, we found no robust evidence for an effect of neuroticism on smoking cessation when using the IVW approach, or when performing sensitivity analyses (Table 3, online Supplementary Tables S3 and S4). This remained the case when looking within UKB (OR 0.997, 95% CI 0.99–1.00, *p* = 0.272; Table 3). There was no strong evidence for an effect of extraversion on smoking cessation when using two-sample MR with summary statistics (OR 0.586, 95% CI 0.07–4.76, *p* = 0.387; Table 3). When restricting our analyses to current and former smokers within UKB, we found weak evidence of an effect and the direction of effect was consistent with that shown in the two-sample analysis. Each additional increase in extraversion risk allele was associated with 1.1% lower odds of smoking cessation (95% CI 0.98–1.00, *p* = 0.057; Table 3).

### Sensitivity analyses

Analyses involving neuroticism were also performed restricting to participants whose genetic data were not included in the interim release of UKB data. Full results are reported in online Supplementary Table S5. Results remained largely consistent with those calculated in the full sample. In this subset, the strength of evidence for the effect of neuroticism on smoking heaviness was weakened (current smokers: *β* = 0.053, 95% CI −0.01 to 0.12, *p* = 0.120). However, the effect size remained consistent, so this may be due to a lack of power in this smaller sample.

IVW effect estimates were calculated for the effect of neuroticism on smoking behaviours using the full release of UKB (online Supplementary Table S6). The effect estimates are consistent with those from the two-sample MR and the one-sample analyses. We find no robust evidence of an association with either smoking initiation or cessation, but strong evidence of an association between neuroticism and increased smoking heaviness.

### Power calculations

We performed power calculations for the two-sample MR analyses. Assuming that the genetic instrument for personality traits explains around 1% of the variation in the exposure and that our sample comprises of around 50% ever smokers, our sample had approximately 86% power to detect 25% greater odds of smoking initiation per each s.d. increase in personality trait. We had around 83% power to detect a change of 0.15 cigarettes per day or 25% lower odds of smoking cessation per s.d. increase in personality trait.

## Discussion

We attempted to disentangle the relationship between smoking and the personality traits of neuroticism and extraversion. We found evidence of a modest genetic correlation with both neuroticism and extraversion, which could indicate a causal pathway between personality and smoking behaviours. However, it is not clear in which direction this pathway acts, and there are also alternative explanations such as horizontal pleiotropy (direct genetic effects on multiple traits) (Davey Smith and Hemani, 2014). We used one- and two-sample MR analyses to investigate these pathways in more detail.

Given that available GWAS summary statistics for neuroticism and extraversion are not stratified by smoking status, we initially used two-sample MR approaches to look at the bidirectional association with smoking initiation. This was followed by one-sample analyses using individual-level data from UKB, in instances where these data were available. When looking at the effect of smoking on personality, we found weak evidence that the rs6265 variant for smoking initiation was associated with both lower neuroticism and higher extraversion. When looking in the reverse direction, evidence was stronger. We found some evidence that each additional neuroticism effect allele was associated with smoking an additional 0.07 cigarettes per day, and that each additional extraversion effect allele was associated with 1.5% greater odds of ever smoking. There was also some suggestion that the extraversion risk score was associated with lower smoking cessation, although the evidence for this was weak.

Estimates from both two-sample MR and one-sample analyses in UKB found a consistent direction of effect, with the smoking initiation instrument (rs6265) associated with a reduction in neuroticism scores, although the evidence for this was weak across both analyses. Although this is a strong instrument for smoking initiation, there could be potential horizontal pleiotropic effects. A recent GWAS investigating risk-taking behaviours used a multi-trait analysis of GWAS approach and identified the rs6265 variant as a lead SNP associated with general-risk-tolerance (Linnér *et al*., 2018). One approach to reduce the potential impact of these effects could be to identify additional strongly associated, but independent variants to include in the smoking initiation instrument by relaxing the *p* value threshold used to select instruments from the original GWAS, as all horizontal pleiotropic effects would need to operate in the same direction (Gage *et al*., 2017).

Analyses from personality to smoking behaviours found some evidence of an association between higher neuroticism and smoking heaviness using one-sample MR and two-sample MR after adjusting for horizontal pleiotropy. Both the observational and MR analyses found a stronger effect among current smokers. These findings are consistent with the self-medication hypothesis (Khantzian, 1985, 1997), and in line with previous literature, such as a twin study by Kendler *et al*. which identified an association with smoking and nicotine dependence (Kendler *et al*., 1999). It is also possible that this pathway is causal only among smokers who find it hard to quit. Observationally, we see higher levels of neuroticism among current smokers and stronger evidence of a genetic correlation among this subgroup of individuals, the divergent effects shown in the one-sample MR would be consistent with this interpretation. However, if both neuroticism and smoking heaviness are associated with difficulty in quitting, then it is possible that this association is due to collider bias. These findings could be used to inform existing smoking cessation services, with the type or intensity of intervention tailored according to an individual's level of neuroticism.

We also observed some evidence of an association between extraversion and smoking. Unlike neuroticism, extraversion did not show evidence of a causal relationship with smoking heaviness, but we did find an association with smoking initiation. Although there was no strong evidence of an association when using a two-sample approach, this could be due to a lack of power given that the direction of effect was consistent with that observed in UKB. Using UKB data, there was evidence that individuals with a higher genetic liability for extraversion had greater odds of taking up smoking. One potential mechanism for this is that extraversion could lead to more social contacts and greater susceptibility to peer influences, which are known to be important in smoking initiation (Kobus, 2003; Haas and Schaefer, 2014). These findings could be used to improve existing smoking prevention schemes, for example, tailoring the messages and images used in health warnings.

There are a number of limitations to our analysis that should be considered. First, UKB formed a large part of the discovery cohort for the GWAS of neuroticism. We were therefore unable to use weighted risk scores to assess the association between smoking phenotypes and neuroticism in our one-sample analyses – weights should be identified in independent samples to avoid overfitting the data and introducing bias into effect estimates (Hartwig and Davies, 2016). However, we performed sensitivity analyses restricting to individuals who were not included in the discovery samples as well as estimating IVW estimates, and results remained consistent. Second, we were unable to use two-sample methods to assess the association from smoking heaviness and cessation to neuroticism and extraversion because the personality summary statistics were not stratified by smoking status. However, we did investigate the association in both directions for neuroticism when using the UKB data. Both the two-sample and UKB analyses gave consistent results when looking at the neuroticism to smoking initiation relationship. Third, due to the lack of an extraversion phenotype currently available in UKB, we were unable to investigate whether there was evidence of an effect from smoking to extraversion. Fourth, MR analyses can often suffer from a lack of power, with large sample sizes and strong instruments required to detect effects. We have identified genetic variants robustly associated with each trait of interest based on results of large recently published GWAS in order to maximise the strength of our instruments. In this analysis, we have triangulated our findings using a combination of one- and two-sample MR approaches to maximise our power to detect any effect (Lawlor *et al*., 2017). Fifth, we stratified on smoking status to investigate the association of smoking heaviness and cessation phenotypes. Although this allows us to investigate pleiotropy, there is the potential to introduce collider bias when stratifying on an exposure (Munafò *et al*., 2018). However, in these stratified analyses, our instruments are principally associated with smoking heaviness and cessation rather than smoking status, so that the risk of collider bias is minimised (Gage *et al*., 2016).

In conclusion, we found evidence of modest genetic correlation between smoking behaviour and both neuroticism and extraversion. We found some evidence for specific causal pathways from personality to smoking phenotypes – from higher neuroticism to heavier cigarette consumption, and from higher extraversion to greater odds of smoking initiation. Evidence in the alternative direction, suggesting that smoking behaviour influences personality, was weaker. These findings could be used to inform future smoking interventions, for example, tailoring existing smoking cessation schemes to provide people with high or low neuroticism a different type or intensity of service.

## Supplementary material

For supplementary material accompanying this paper visit https://doi.org/10.1017/S0033291718003069. click here to view supplementary material
