# Supplementary material for: Cigarette smoking and personality: interrogating causality using Mendelian randomisation
Source: Psychol Med. 2018 Oct 25;49(13):2197–205. doi: 10.1017/S0033291718003069 (PMC6747344; doi:10.1017/S0033291718003069)
Supplement: Supplementary file 1 [file S0033291718003069sup001.docx]

**Supplementary Material**

Table S1. Genetic variants included in the neuroticism and extraversion scores, and their association with each phenotype.

|  |  | |  | |  | |  | | SNP-personality trait | | | | | SNP-smoking initiation | | | SNP-heaviness | | | SNP-cessation | | |
| --- | --- | --- | --- | --- | --- | --- | --- | --- | --- | --- | --- | --- | --- | --- | --- | --- | --- | --- | --- | --- | --- | --- |
| SNP | Original SNP if proxy used in analysis (r^2^) | | Chr | | A1/A2 | | EAF | | β | | SE | | P | log(OR) | SE | P | log(OR) | SE | P | log(OR) | SE | P |
| Neuroticism |  | |  | |  | |  | |  | |  | |  |  |  |  |  |  |  |  |  |  |
| rs6787412 | rs35688236 (0.861) | | 3 | | A/G | | 0.623 | | 0.017 | | 0.004 | | 1.6x10^-6^ | -0.002 | 0.012 | 0.843 | -0.077 | 0.084 | 0.361 | -0.023 | 0.016 | 0.169 |
| rs2572431 |  | | 8 | | T/C | | 0.595 | | 0.028 | | 0.003 | | 4.2x10^-16^ | 0.001 | 0.012 | 0.909 | 0.062 | 0.082 | 0.452 | 0.010 | 0.016 | 0.538 |
| rs10960067 | rs10960103 (0.925) | | 9 | | A/G | | 0.780 | | 0.025 | | 0.004 | | 8.8x10^-10^ | -0.009 | 0.017 | 0.598 | -0.080 | 0.115 | 0.489 | -0.015 | 0.022 | 0.476 |
| rs2150462 |  | | 9 | | G/C | | 0.259 | | 0.022 | | 0.004 | | 2.7x10^-8^ | -0.023 | 0.013 | 0.084 | 0.040 | 0.091 | 0.657 | 0.011 | 0.018 | 0.532 |
| rs1822293 | rs139237746  (0.87) | | 11 | | A/G | | 0.459 | | 0.017 | | 0.003 | | 4.4x10^-7^ | 0.003 | 0.012 | 0.825 | -0.230 | 0.081 | 0.005 | -0.001 | 0.016 | 0.972 |
| rs4938021 |  | | 11 | | T/C | | 0.621 | | 0.023 | | 0.004 | | 4.0x10^-10^ | 0.006 | 0.012 | 0.640 | 0.088 | 0.084 | 0.292 | -0.005 | 0.017 | 0.752 |
| rs12903563 |  | | 15 | | T/C | | 0.494 | | 0.020 | | 0.004 | | 2.9x10^-8^ | 0.029 | 0.012 | 0.018 | -0.047 | 0.086 | 0.586 | -0.027 | 0.017 | 0.110 |
| rs12938775 | | 17 | | G/A | | 0.463 | | 0.020 | | 0.004 | | 8.5x10^-9^ | | 0.031 | 0.031 | 0.318 | -0.177 | 0.220 | 0.420 | 0.029 | 0.046 | 0.526 |
| rs193236081 | No proxy | | 17 | | G/T | | 0.269 | | 0.027 | | 0.004 | | 6.3x10^-11^ | - | - | - | - | - | - | - | - | - |
| rs11082011 | rs1557341  (1.00) | | 18 | | C/T | | 0.369 | | 0.021 | | 0.004 | | 8.6x10^-9^ | 0.0089 | 0.021 | 0.675 | 0.061 | 0.129 | 0.636 | -0.019 | 0.025 | 0.434 |
| rs12961969 |  | | 18 | | A/C | | 0.218 | | 0.024 | | 0.004 | | 2.2x10^-8^ | 0.014 | 0.016 | 0.357 | 0.202 | 0.108 | 0.060 | 0.001 | 0.021 | 0.954 |
| Extraversion |  | |  | |  | |  | |  | |  | |  |  |  |  |  |  |  |  |  |  |
| rs57590327 | No proxy | | 3 | | T/G | | 0.287 | | 0.026 | | 0.006 | | 2.0x10^-5^ | - | - | - | - | - | - | - | - | - |
| rs2164273 |  | | 8 | | G/A | | 0.426 | | 0.024 | | 0.006 | | 4.1x10^-5^ | 0.007 | 0.012 | 0.567 | 0.011 | 0.084 | 0.898 | -0.010 | 0.016 | 0.546 |
| rs2045147 | rs6481128 | | 10 | | A/G | | 0.444 | | 0.018 | | 0.005 | | 6.6x10^-4^ | 0.019 | 0.012 | 0.105 | 0.096 | 0.082 | 0.244 | -0.023 | 0.016 | 0.152 |
| rs3764002 | rs1426371 | | 12 | | C/T | | 0.285 | | 0.022 | | 0.006 | | 2.8x10^-4^ | 0.011 | 0.022 | 0.624 | -0.247 | 0.137 | 0.071 | 0.009 | 0.026 | 0.742 |
| rs7498702 | No proxy | | 16 | | T/C | | 0.317 | | 0.026 | | 0.006 | | 1.2x10^-5^ | - | - | - | - | - | - | - | - | - |

Table S2. Amount of variation in neuroticism and smoking behaviours explained by each instrument in UK Biobank

| **Exposure** | **Instrument** | **F-statistic** | **R^2^** |
| --- | --- | --- | --- |
| **Neuroticism** | 10 SNPs from Okbay et al. (2016) | 261.4 | 0.002 |
| **Extraversion** | 5 SNPs from Lo et al. (2017) | - | - |
| **Smoking initiation** | rs6265 | 27.6 | 0.001 |
| **Former smoking heaviness** | rs16969968 | 215.6 | 0.039 |
| **Current smoking heaviness** | rs16969968 | 92.7 | 0.040 |
| **Smoking cessation** | rs3025343 | 41.9 | 0.0004 |

Table S3. Estimates from two-sample MR sensitivity analyses of neuroticism and smoking behaviours

| **Exposure** | **Outcome** | **Instrument** | **Analysis** | **Effect size** | **95% CI** | **P-value** |
| --- | --- | --- | --- | --- | --- | --- |
| **Neuroticism** | **Smoking initiation** | 10 SNPs from Okbay et al. (2016) | IVW | 0.153 | -0.34, 0.65 | 0.499 |
|  |  |  | MR-Egger – slope | -0.406 | -3.55, 2.74 | 0.772 |
|  |  |  | MR-Egger - pleiotropy | 0.012 | -0.06, 0.08 | 0.687 |
|  |  |  | Weighted median | 0.118 | -0.42, 0.66 | 0.676 |
|  |  |  | Penalised weighted median | 0.109 | -0.43, 0.65 | 0.699 |
|  | **Smoking heaviness** | 10 SNPs from Okbay et al. (2016) | IVW | 0.050 | -4.11, 4.21 | 0.979 |
|  |  |  | MR-Egger – slope | 22.55 | 2.82, 42.27 | 0.027 |
|  |  |  | MR-Egger - pleiotropy | -0.500 | -0.93, -0.07 | 0.026 |
|  |  |  | Weighted median | 1.992 | -1.72, 5.70 | 0.330 |
|  |  |  | Penalised weighted median | 2.075 | -1.60, 5.75 | 0.311 |
|  | **Smoking cessation** | 10 SNPs from Okbay et al. (2016) | IVW | -0.227 | -0.84, 0.39 | 0.423 |
|  |  |  | MR-Egger – slope | 1.819 | -1.95, 5.56 | 0.294 |
|  |  |  | MR-Egger - pleiotropy | -0.046 | -0.13, 0.04 | 0.236 |
|  |  |  | Weighted median | -0.062 | -0.78, 0.65 | 0.868 |
|  |  |  | Penalised weighted median | -0.062 | -0.78, 0.65 | 0.864 |

Table S4. Tests of heterogeneity in the SNP-neuroticism association

| **Exposure** | **Outcome** | **Instrument** | **Method** | **Q** | **df** | **P-value** |
| --- | --- | --- | --- | --- | --- | --- |
| **Neuroticism** | **Smoking initiation** | 10 SNPs from Okbay et al. (2016) | IVW | 10.65 | 9 | 0.300 |
|  |  |  | MR-Egger | 10.43 | 8 | 0.236 |
|  |  |  | Q’ | 0.23 | 1 | 0.634 |
|  | **Smoking heaviness** | 10 SNPs from Okbay et al. (2016) | IVW | 15.87 | 9 | 0.070 |
|  |  |  | MR-Egger | 8.40 | 8 | 0.395 |
|  |  |  | Q’ | 7.47 | 1 | 0.006 |
|  | **Smoking cessation** | 10 SNPs from Okbay et al. (2016) | IVW | 6.12 | 9 | 0.728 |
|  |  |  | MR-Egger | 4.51 | 8 | 0.809 |
|  |  |  | Q’ | 1.61 | 1 | 0.204 |

Note: df = degrees of freedom where degrees of freedom is equal to the number of SNPs -1. Q = Rucker’s Q, a test of heterogeneity or dispersion in the SNP-exposure effects.

The Q’ indicates the extent to which MR Egger is a better fit than the inverse-variance weighted method.

Table S5. Two-sample bidirectional MR estimates of smoking phenotypes and personality traits restricting to participants whose genetic data was not included in the interim release.

| **Exposure** | **Outcome** | **Instrument** | **Sample** | **Effect size** | **95% CI** | **P-value** | **N** |
| --- | --- | --- | --- | --- | --- | --- | --- |
| **Smoking initiation** | **Neuroticism** | rs6265 | All | -0.026 | -0.05, 6.9x10^-5^ | 0.051 | 196,769 |
| **Smoking heaviness** | **Neuroticism** | rs16969968 | Non-smokers | 0.008 | -0.02, 0.04 | 0.588 | 108,259 |
|  |  |  | Former smokers | 0.009 | -0.03, 0.05 | 0.615 | 70,326 |
|  |  |  | Current smokers | 0.021 | -0.06, 0.10 | 0.587 | 17,626 |
| **Smoking cessation** | **Neuroticism** | rs3025343 | Non-smokers | -0.005 | -0.05, 0.04 | 0.816 | 108,259 |
|  |  |  | Ever smokers | 0.015 | -0.03, 0.06 | 0.554 | 87,952 |
| **Neuroticism** | **Smoking initiation** | Unweighted score | All | 1.000 | 0.996, 1.004 | 0.920 | 229,062 |
| **Neuroticism** | **Smoking heaviness** | Unweighted score | Non-smokers | - | - | - | - |
|  |  |  | Former smokers | -0.012 | -0.05, 0.03 | 0.583 | 48,900 |
|  |  |  | Current smokers | 0.053 | -0.01, 0.12 | 0.120 | 13,109 |
| **Neuroticism** | **Smoking cessation** | Unweighted score | Ever smokers | 0.994 | 0.99, 1.00 | 0.092 | 102,636 |

Table S6. IVW estimates of neuroticism to smoking phenotypes in UK Biobank

| **Exposure** | **Outcome** | **Instrument** | **Sample** | **Effect size** | **95% CI** | **P-value** |
| --- | --- | --- | --- | --- | --- | --- |
| **Neuroticism** | **Smoking initiation** | Unweighted score | All | 1.338 | 0.30, 5.97 | 0.703 |
| **Neuroticism** | **Smoking heaviness** | Unweighted score | Non-smokers | - | - | - |
|  |  |  | Former smokers | 0.500 | 0.31, 0.68 | <0.001 |
|  |  |  | Current smokers | 0.416 | 0.29, 0.54 | <0.001 |
| **Neuroticism** | **Smoking cessation** | Unweighted score | Ever smokers | 1.239 | 0.45, 3.44 | 0.982 |
